# Analysis of *PRX* Gene Family and Its Function on Cell Lignification in Pears (*Pyrus bretschneideri*)

**DOI:** 10.3390/plants10091874

**Published:** 2021-09-10

**Authors:** Zhihua Xie, Weikang Rui, Yazhou Yuan, Xiaofei Song, Xing Liu, Xin Gong, Jianping Bao, Shaoling Zhang, Khanizadeh Shahrokh, Shutian Tao

**Affiliations:** 1 Pear Engineering Research Centre, College of Horticulture, Nanjing Agricultural University, 1 Weigang, Nanjing 210095, China; xzz811@126.com (Z.X.); ruiweikang@njau.edu.cn (W.R.); 2016104031@njau.edu.cn (Y.Y.); 2015104031@njau.edu.cn (X.S.); liuxing4834@126.com (X.L.); 2016104030@njau.edu.cn (X.G.); zhangsl@njau.edu.cn (S.Z.); 2 College of Plant Science, Tarim University, Ala’er 843300, China; baobao-xinjiang@126.com; 3 ELM Consulting Inc., St-Lazare, QC J7T 3C2, Canada; Shahrokh.Khanizadeh@outlook.com

**Keywords:** class III peroxidases, genome-wide, stone cells, lignification, expression patterns, verification, pear

## Abstract

Class III peroxidases (PRXs) are plant-specific enzymes that play key roles in the responses to biotic and abiotic stress during plant growth and development. In addition, some peroxidases also play roles in plant lignification. In this study, a total of 114 *PRX* (designated *PbPRXs*) genes were identified in the pear (*Pyrus bretschneideri Rehd*) genome based on systematic analysis. These *PRX* genes were divided into 12 groups based on their phylogenetic relationships. We performed systematic bioinformatics analysis of the *PRX* genes, including analysis of gene structures, conserved motifs, phylogenetic relationships, and gene expression patterns during pear fruit growth. The *PbPRXs* are unevenly distributed on the 17 pear chromosomes and some of them on other scaffolds. Gene duplication event analysis indicated that whole-genome duplication (WGD) and segmental duplication play key roles in *PRX* gene amplification. Ka/Ks analysis suggested that most duplicated *PbPRXs* experienced purifying selection, with limited functional divergence during the duplication events. Furthermore, the analysis indicated that those highly expressed genes might play significant roles in the lignification of cells to form stone cells in pear fruit. We examined the expression of those highly expressed genes during fruit growth using quantitative real-time PCR (qRT-PCR), verifying differential expression patterns at different stages of fruit. This study provides useful information for further functional analysis of the *PRX* gene family in pears.

## 1. Introduction

Class III peroxidases (PRXs; EC 1.11.1.7) are plant-specific enzymes that can catalyze the reduction of H_2_O_2_ by moving electrons to various donor molecules such as phenolic compounds, lignin precursors, or secondary metabolites [1]. Plant PRX proteins are involved in several important physiological and developmental processes, including lignin and suberin formation, the cross-linking of cell wall components, wound healing, the removal of H_2_O_2_, the oxidation of toxic reductants, and defense against pathogen or insect attack [1,2,3]. For example, Arabidopsis peroxidase 72 (*AtPrx72*), which is homologous to *ZePrx*, plays an important role in lignification [4]. Besides *AtPrx72*, *AtPrx02*, *AtPrx09*, *AtPrx17*, *AtPrx30,* and *AtPrx37* are involved in Monolignol polymerization, *AtPrx53* and *AtPrx66* are involved in the lignification of vascular bundles [1]. PRXs include all secretory plant-specific peroxidases [1,5,6], which comprise large multigene families in many plants, such as *Arabidopsis thaliana*, *Oryza sativa,* and *Populus trichocarpa*, with 73, 138, and 93 *PRX*s, respectively [7,8,9].

Pear is one of the most important commercial fruits and is cultivated in all temperate-zone countries of both hemispheres. Stone cell content is an important factor affecting fruit quality. If the content of stone cells is higher, the rougher the fruit flesh will be. Therefore, a decrease of stone cell content is directly related to the improvement of fruit quality.

Unfortunately, most previous studies focused on cultivation, such as bagging and pruning, but this view in its nature is still lacking. As we all know, lignin is the main component of stone cells [10,11]. Thus, identification of important genes related to lignin formation and accumulation, and understanding their functional mechanism, will help reduce lignin content in pears, thus improve the quality of the pear.

However, peroxidases, which may be involved in lignin accumulation, have little been reported in pears.

Due to the fast development of sequencing techniques, more and more genomes in plants have been sequenced. In the past few years, repeated episodes of small-scale and large-scale gene duplication events have been shown to play important roles during the evolution of gene families. Large-scale gene duplication includes segmental duplications and whole-genome duplications (WGDs) [12]. In pears, evidence has indicated that two WGDs occurred during pear genome evolution, with an ancient WGD event ~140 million years (Myr) [13], and a recent WGD event at 30–45 Myr [14]. Small-scale gene duplication events, such as tandem duplications, also play important roles during gene family expansion [15]. The sum of other small-scale duplications and tandem duplications are estimated to contribute duplicates on a scale comparable to large segmental duplications in rice [16]. The evidence has indicated that tandem and segmental duplications are important during gene family expansion [17,18].

Recently, the pear (*Pyrus bretschneideri Rehd*) genome was also sequenced and assembled by the strategy of BAC by BAC, combined with whole-genome shotgun data, a total of 194x genome coverage sequencing. The high quality of the assembled sequence and annotation were assessed and confirmed using Sanger-derived BAC sequences along with RNA-seq of different tissues and public protein database alignment [19]. The high quality of the pear genome is suitable for genome-wide identification and analysis of gene families.

The present study is the first to report on the genome-wide identification of class III peroxidase genes in pear, although they have been identified from other ligneous or herbaceous species, together with phylogenetic, structural, and evolutionary analysis. In addition, RNA-seq databases of pear fruit were used to determine the expression pattern for all *PRX* genes and select key genes affecting lignin formation. This study will help to reveal the roles of these *PRX* genes in pear stone cell formation as well as provide gene resources for the future genetic improvement of pears. The obtained results will also provide a reference on lignin formation for other related plants.

## 2. Materials and Methods

### 2.1. Identification of Class III Peroxidase Genes (PRXs) in Pear

To identify members of the class III peroxidase gene family, multiple database searches were performed. The Arabidopsis class III peroxidase (AtPRXs) gene sequences obtained from the TAIR database were used as queries to perform repetitive blast searches against the Pear Center (http://peargenome.njau.edu.cn/) [19]. Additionally, all protein sequences were then used as queries to perform multiple database searches against proteome and genome files downloaded from these databases. Stand-alone versions of BLASTP and TBLASTN available from the Basic Local Alignment Search Tool were used with the e-value cutoff set to 1 × 10^−3^. All retrieved nonredundant sequences were collected from the phytozome database v9.1, and subjected to domain analysis by using two different domain analysis programs: the Pfam 27.0 and SMART, with the default cut off parameters [20,21]. Genes without PRX-specific peroxidase domains were rejected.

### 2.2. Phylogenetic Analysis, Gene Structure and Conserved Motif Analysis

The PRX family protein sequence alignments and the phylogenetic tree were created by using the Muscle program. The phylogenetic trees for pear class III peroxidase genes were constructed using the maximum likelihood (ML) method in MEGA6.0 and assessed by bootstrap analysis with 1000 resampling replicates.

To determine the exon/intron structures of the *PbPRX* genes, the Gene Structure Display Server (GSDS) (http://gsds.cbi.pku.edu.cn/) [22] was used to align their cDNAs with the corresponding genomic DNA sequences. The conserved motifs were detected using the online MEME (Multiple Expectation Maximization for Motif Elicitation) tool (http://meme.sdsc.edu/meme430/intro.html) [23]. Parameters were set as follows: number of repetitions; optimum motif width set to ≥6 and ≤200; maximum number of motifs set to 20. The conserved motifs were analyzed with the SMART (http://smart.embl-heidelberg.de/) and Pfam (http://pfam.sanger.ac.uk/search) programs.

### 2.3. Chromosomal Location and Synteny Analysis

The chromosomal location information of the *PbPRX* genes was obtained from genome annotations. The data were then displayed by using Circos. The analysis of synteny in the pear genomes was conducted locally by using a method similar to that developed for the PGDD (http://chibba.agtec.uga.edu/duplication/) [24]. Duplications of *PbPRX*s were identified using MCScanX software (http://chibba.pgml.uga.edu/mcscan2/) [25]. First, BLASTP was performed to search for potential paralogy gene pairs (E < 1 × 10^−5^, top 5 matches) genomes. Then, these paralogy pairs were used as the input for MCScanX to identify syntenic chains [25]. MCScanX was further used to identify WGD, segmental, tandem duplications in the *PbPRX* gene family.

### 2.4. Calculating Ka and Ks of the PbPRX Gene Family

The valid gene pairs derived from different gene duplication modes were used to calculate the nonsynonymous (Ka) and synonymous (Ks) substitution rates. KaKs Calculator 2.0 software with default parameters was used to calculate Ka and Ks values, and Ka/Ks ratios based on a model-averaged method [26].

Ka/Ks calculation was applied to estimate the selection pressure of *PRX* gene pairs. The algorithm was NG.

### 2.5. Gene Ontology Enrichment Analysis

InterPro domains were annotated by InterProScan [27] Release 36.0 and functional assignments were mapped onto Gene Ontology (GO) [28]. Furthermore, the GO classification and draw GO tree using WEGO [29].

### 2.6. Genome-Wide Expression Analysis of PRX Gene Family

To investigate the expression of *PRX* gene family members, pear fruit samples of the ‘Dangshansuli’ cultivar on 22 April (15 days after full bloom, DAFB), 13 May (36 DAFB), 27 June (81 DAFB), 28 July (110 DAFB), and 30 August (145 DAFB) were collected in 2011, which included the key stages of pear fruit development from early fruit setting to mature. RNA sequencing libraries of five fruit developmental stages were constructed using an Illumina standard mRNA-Seq Prep Kit (TruSeq RNA and DNA Sample Preparation Kits version 2). The RNA-seq data was downloaded from our center website (http://peargenome.njau.edu.cn/). Expression values of each gene were logarithm, the cluster analyses were performed using cluster software with the hierarchical cluster method of “complete linkage” and Euclidean distances. Finally, the Treeview program was used to display the results of the cluster.

### 2.7. RNA Extraction and First-Strand cDNA Synthesis

In our research, four fruit stages were sampled depending on the status of pear development in 2016, 25 April (21 DAFB), 17 May (42 DAFB), 28 June (84 DAFB), and 26 July (112 DAFB) for quantitative real-time PCR (qRT-PCR) analysis. Total genomic RNA was extracted from the pear fruit using the Plant Total RNA Isolation Kit Plus (FOREGENE CO.,LTD, China). A260/A280 ratios of the RNA ranged from 1.9 to 2.1 quantified with a NanoDrop ND1000 spectrophotometer. Finally, about 2 μg of total RNA was used for first-strand cDNA synthesis using REVERTAID 1ST CDNA SYNTH KIT (Fermentas Co.,Ltd, Lithuania) according to the manufacturer’s protocol.

### 2.8. Quantitative Real-Time PCR Analysis

The primers used for amplifying *PRX* genes are listed in Supplementary Table S1. In the present study, the LightCycler 480 SYBR GREEN I Master (Roche, Nutley, NJ, USA) was used according to the manufacturer’s protocol. Each reaction mixture contained 10 μL of LightCycler 480 SYBR GREEN I Master, 0.4 μL of each primer, 1 μL of diluted cDNA and 7.4 μL nuclease-free water. The qRT-PCR was performed on the LightCycler 480 (Roche, USA) and all reactions were run as duplicates in 96-well plates. The qRT-PCR reaction conditions were as follows: pre-incubation at 95 °C for 10 min and then 40 cycles of 94 °C for 15 s, 60 °C for 30 s, 72 °C for 30 s, and finally, extension at 72 °C for 3 min, and reading the plate for fluorescence data collection at 60 °C. A melting curve was performed from 60 °C to 95 °C in order to check the specificity of the amplified product. The real-time PCR experiment was carried out three times under identical conditions. Finally, the average threshold cycle (Ct) was calculated per sample, *Pyrus* Actin was used as the internal control, and the relative expression levels were calculated with the 2^−ΔΔCt^ method described by Livak et al. [30].

### 2.9. Determination of Stone Cells and Lignin Content

Each fruit was peeled, cored, and diced into cubes. A 100 g sample of pear flesh was stored in the refrigerator at −20 °C for at least 24 h then homogenized with distilled water in a blender for 10 min. The homogenate was then diluted with distilled water. The suspension was incubated at room temperature for 30 min and the supernatant phase was decanted. Finally, the sediment was suspended in 0.5 M HCl for 30 min, decanted, and washed with distilled water. This operation was repeated several times until the stone cells were almost free of extraneous cell debris [31].

The method was carried out as described by Tao et al. [10] with some modifications. Pear flesh was dried in an oven at 65 °C. The dry pear flesh was ground and pestled in 95% ethanol, then the sediment was washed with 95% ethanol and ethanol: hexane (1:2, *v/v*) three times, respectively, and dried. Dried sediments were digested in 2 mL of 25% (*v/v*) acetyl bromide in acetic acid and incubated for 30 min at 70 °C. The reaction was terminated by adding 0.9 mL of 2 M NaOH with an extra 5 mL of acetic acid and 0.1 mL of 7.5 M hydroxylamine hydrochloride. The volume was corrected to 10 mL with acetic acid and the absorbance at A280 was measured. The amount of lignin was calculated from a linear calibration curve with commercial alkali lignin (Sigma–Aldrich, St. Louis, MO, USA).

## 3. Results

### 3.1. Identification and Construction of Phylogenetic Tree of Class III Peroxidase Gene Family (PRXs) in Pear

In the present study, a total of 114 open reading frames (ORFs) encoding putative PRX proteins were identified in the pear (*Pyrus bretschneideri*)(cultivar: ‘Dangshansuli’) genome using the HMMER profile and BLASTp search for further analysis.

Originally, a total of 126 candidate *PRX* genes were identified in pears. Among these, 12 nontargeted or overlapping protein sequences were manually removed. The results show that all 114 putative pear *PRX* genes contain a conserved PRX domain; this number is greater than that in Arabidopsis (73) [9]. Finally, on the basis of previous research in Arabidopsis, we assigned names to these *PRX* genes (*PbPRX1*–114) according to their chromosomal positions for convenience. The length of the 114 newly identified PRX proteins varies from 84 to 1315 aa, with an average of 336 aa. Other information about the clone number, chromosomal location, molecular weight (Mw), isoelectric point (PI), and exon number of each *PbPRX* gene/protein is listed in Table 1.

**Figure 1:**
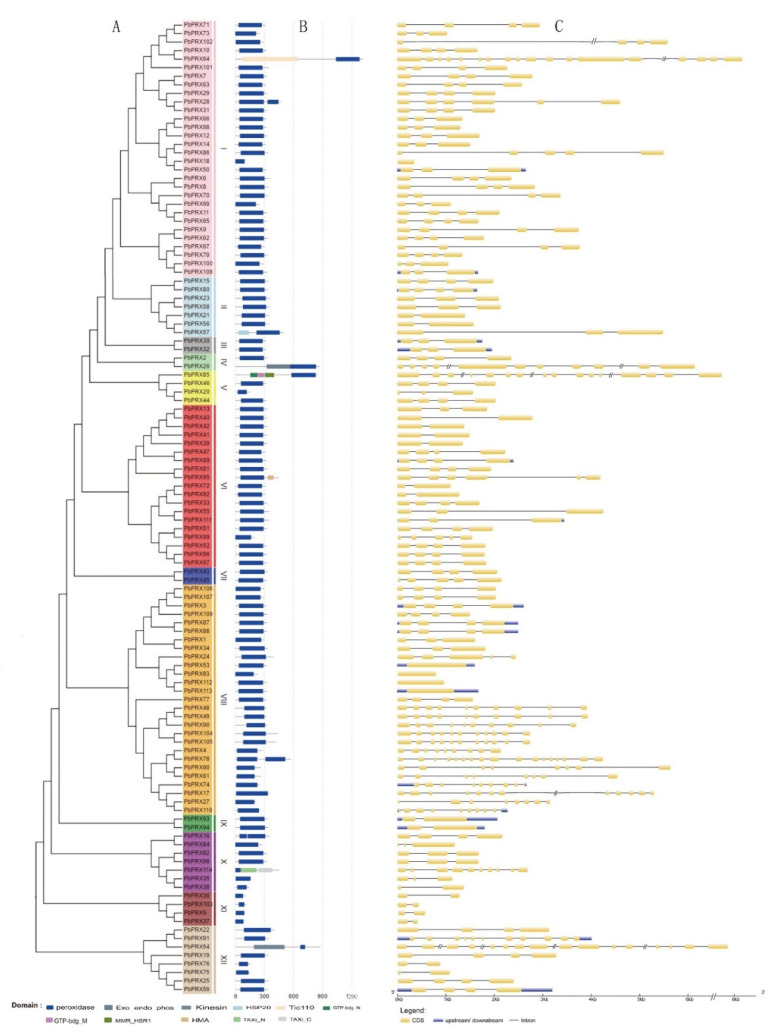
Phylogenetic relation, domain, and gene structure of *PbPRX* gene family. (**A**) Phylogenetic tree of the *PbPRX* gene family. (**B**) Domain information of members of *PbPRX* gene family; (**C**) Exon–intron structure of pear *PRX* genes. Yellow boxes indicate exons; lines indicate introns; blue boxes indicate the upstream or downstream.

To gain insight into the structure of the *PRX* genes, the exon and intron boundaries, which are known to play crucial roles in the evolution of multiple gene families, were analyzed. Results showed that exon numbers of 114 *PRX* genes ranged from one to eighteen (Figure 1C). Different subfamilies contained different exon numbers, the fact that the *PbPRX64* gene has 18 exons, and *PbPRX18*, *PbPRX53*, *PbPRX83*, *PbPRX112*, and *PbPRX113* have only one exon, indicates that both exon gain or loss has occurred during the evolution of the *PRX* gene family, which might lead to the functional diversity of closely related *PRX* genes. However, it was found that within each subfamily, genes usually have a similar number of exons.

**Figure 2:**
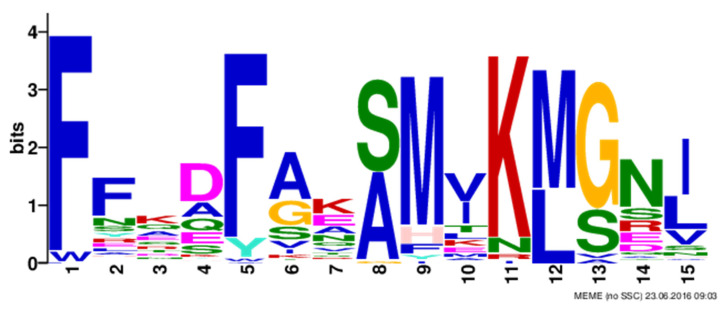
The most motif structure of *PbPRX* gene family.

Phylogenetic analysis of the 114 identified nucleotide sequences of *PbPRX*s could be classified into 12 subfamilies (Figure 2). Group I contains 30 members and group I is the biggest subfamily. Group III, IV, VII, and IX are the smallest subfamilies only containing two members. Across the Maximum Likelihood (ML) tree, most bootstrap values were 80 or higher, and 12 nodes of each subfamily clade had a good bootstrap value.

### 3.2. Analysis of Conserved Motifs and Domain

A total of 20 conserved motifs were identified in the pear PRX proteins. Detailed information about the conserved amino acid sequences and lengths of the 20 motifs is shown in Table 2. The conserved motifs obtained from MEME analysis were annotated using the Pfam and SMART programs. Most of the closely related members have the same motif compositions, suggesting that there are functional similarities between PRX proteins within the same subfamily. Figure 2 depicts the structure diagrams of motif 5, which shows that the structure may be the core structure of the function of the *PRX* gene family in pears. Furthermore, some subfamily-specific motifs with unknown functions were also detected, indicating that these motifs are likely required for subfamily-specific functions. However, some motifs are distributed in nearly every subfamily, although their functions remain unknown (motif 4, 5, 10, 11); these motifs might be important for the functions of PbPRX proteins.

All conserved domains of the *PbPRX* gene family are shown in Figure 1B. All PRX proteins contain one or more PRX domain, and this is one of the main bases of the gene family screening.

### 3.3. Chromosomal Locations, Gene Duplication, and Collinearity Analyzes

To determine the genome organization and distribution of *PbPRX*s on different chromosomes in pears, a chromosome map was constructed. The results show that the 99 *PbPRX* genes are distributed on 17 chromosomes with a nonrandom distribution, and 15 *PbPRX* genes are mapped onto the other 10 scaffolds, as shown in Table 1. Chromosome 3 contains the most *PbPRX* genes (13), followed by chromosome 7 (11) and chromosome 8 (11). By contrast, only one *PbPRX* gene is present on chromosomes 1 and 14. In addition, some chromosomes exhibit a relatively high density of *PbPRX* genes, such as the bottoms of chromosomes 3 and 7 and the top of chromosome 10. Gene duplication, including segmental or whole-genome duplication (WGD) and tandem duplication, is considered to be one of the primary driving forces in the evolution of genomes [32,33]. During the evolution of a gene family, tandem duplication and WGD/segmental duplication play important roles in generating new members. Therefore, in order to clarify the potential mechanism of evolution of the *PRX* gene family, both tandem duplication and segmental duplication events were investigated in this study. In this study, 26 related duplicated gene pairs were identified (Table 3), which cover most of the 30 sister pairs. Among the 114 *PbPRX* genes identified, a total of 26 gene pairs (46 genes) were localized to WGD/segmentally duplicated regions, while there is no gene in tandem repeats. These results indicate that WGD/segmental duplications were the main contributors to the expansion of the pear *PRX* family. To explore the selection pressures among *PbPRX* duplicated genes, we calculated the Ka, Ks, and Ka/Ks values for all the *PRX* gene pairs. The results were not shown except for 26 gene pairs (Table 3). In general, Ka/Ks > 1 indicates positive selection, Ka/Ks = 1 indicates neutral selection, and Ka/Ks < 1 indicates negative or purifying selection. The Ka/Ks ratios of most *PbPRX* gene pairs were <1, suggesting that these gene pairs evolved under purifying selection in pears. The results of this Ka/Ks analysis suggest that negative or purifying selection was vital to the functional divergence of *PbPRX* genes.

### 3.4. Functional Annotation with Gene Ontology

**Figure 3:**
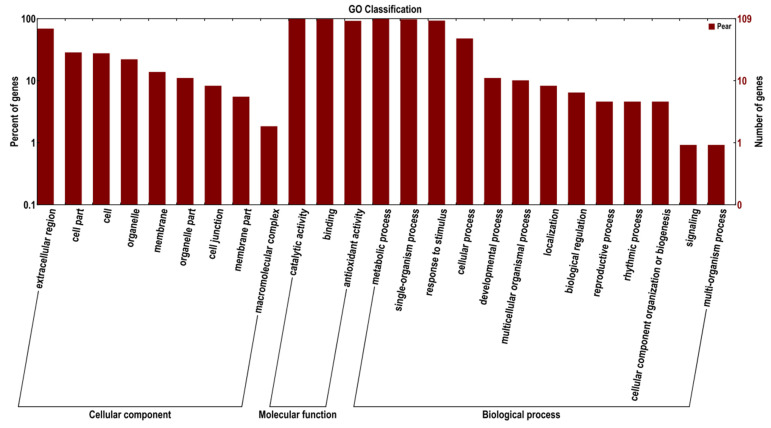
GO categories of the genes identified. Y-axis (**left**) represents percentages of genes identified in this study, Y-axis (**right**) represents the actual gene number. The genes were annotated in three main categories: biological process, cellular component, and molecular function (X-axis).

In this study, a total of 109 differentially expressed genes that could be categorized into 25 functional groups were found (Figure 3). The major subcategories were as follows: one for cellular component (‘extracellular region’); three for molecular function (‘catalytic activity’, ‘binding’, and ‘antioxidant activity’); and three for biological process (‘metabolic process’, ‘single-organism process’, and ‘response to stimulus’). These results indicate that *PbPRX* genes are mainly functioning in ‘catalytic activity’, ‘binding’, ‘metabolic process’.

### 3.5. Expression of the PRX Gene Family in Pears

**Figure 4:**
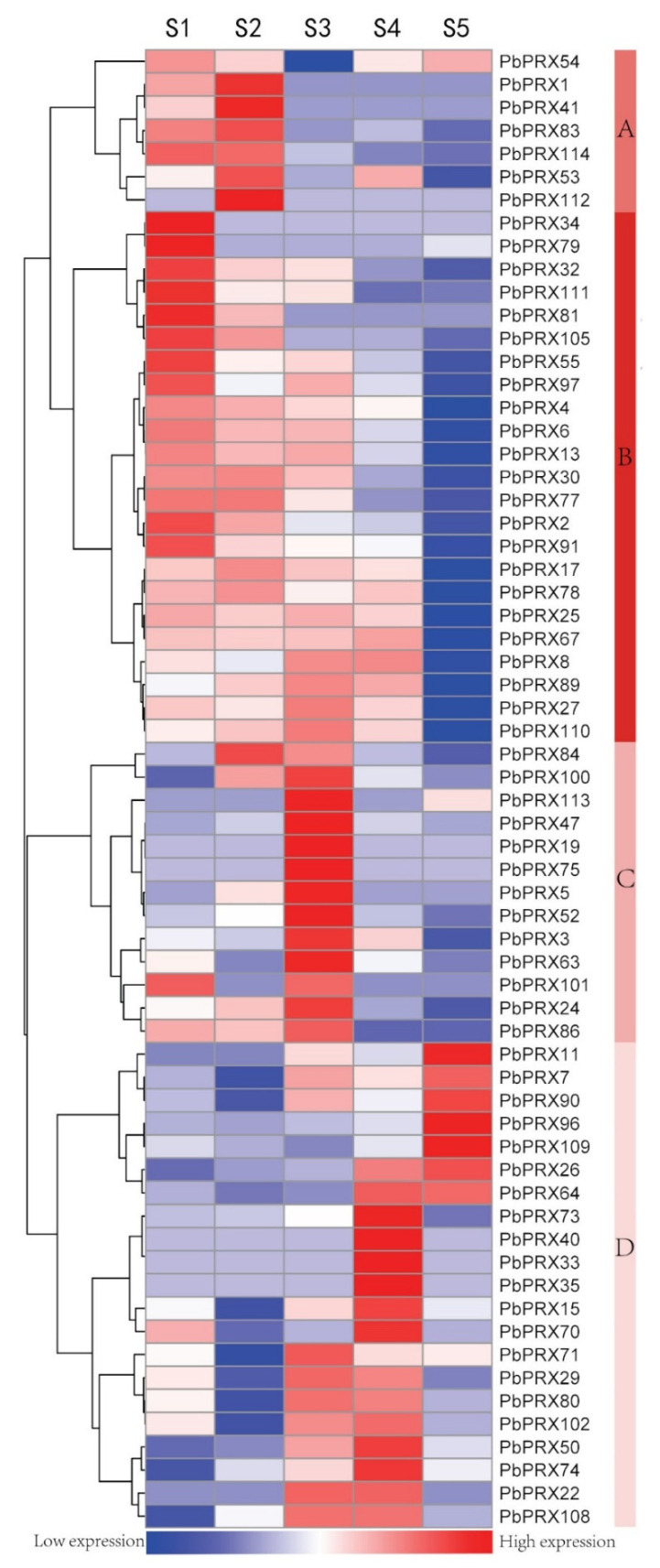
Heat map representation of *PbPRX* genes the five stages of pear fruit development. S1 22 Apr, 15 days after full bloom (DAFB); S2 13 May, 36DAFB; S3 27 June, 81DAFB; S4 28 July 110DAFB; S5 30 Aug, 145DAFB. A B C D on the right means different branches.

To investigate the transcript pattern of *PRX* family genes during fruit development, the expression patterns over six developmental stages of the pear fruit, from the early to mature stage, were analyzed using the RNA-seq database available from our previous research [19]. According to publicly available genome-wide transcript profiling data from pear tissues, of the 114 *PbPRX* genes, only 64 *PbPRX*s are expressed in fruit. Finally, a hierarchical cluster with the logarithm of average values for the 64 *PRX* family members was generated. As shown in Figure 4, *PRX* family genes can be divided into four major groups based on their expression profiles. Group A contained seven *PRX* genes, all of them exhibited preferential expression in the first two stages, indicating that those genes may play important roles in lignin formation during early fruit development. In addition, 23 *PRX* genes belong to group B, which showed high expression in the first three stages, among them, *PbPRX55* and *PbPRX111* had the highest expression levels in the first stage of fruit development. Group C included 13 *PRX* genes, which showed high expression in the S3 stages, but were almost all lower than the expression of group A and B. Group D consisted of 21 *PRX* genes that displayed higher expression in the last three stages, and with low expression during the foregoing stage of fruit development.

### 3.6. Stone Cells and Lignin Content of Pear Fruit during Pear Fruit Development

**Figure 5:**
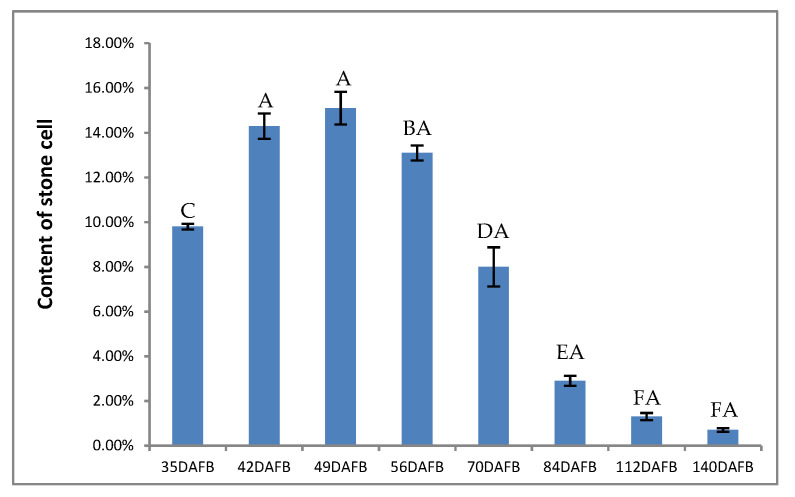
Content of stone cells during pear development. Different big letters indicate significant difference at 0.01 level.

To investigate the formation of stone cells, we determined the content of stone cells from early fruit set to maturity. We found that the content of stone cells reached a maximum at 49 DAFB, and after that, the stone cells number was reduced. The content of stone cells was at a minimum when the fruit was mature (Figure 5).

**Figure 6:**
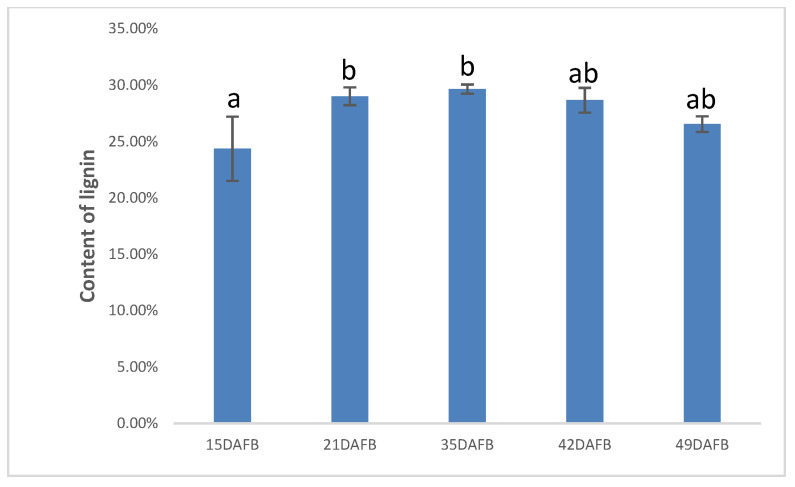
Lignin content in the pulp powder. Different small letters indicate significant difference at 0.05 level.

To investigate the formation of lignin during pear fruit development, we determined the content of lignin in the pulp powder of pear. We found that the content of lignin reached its maximum at 35 DAFB (Figure 6).

### 3.7. Verification of Gene Expression by qRT-PCR

**Figure 7:**
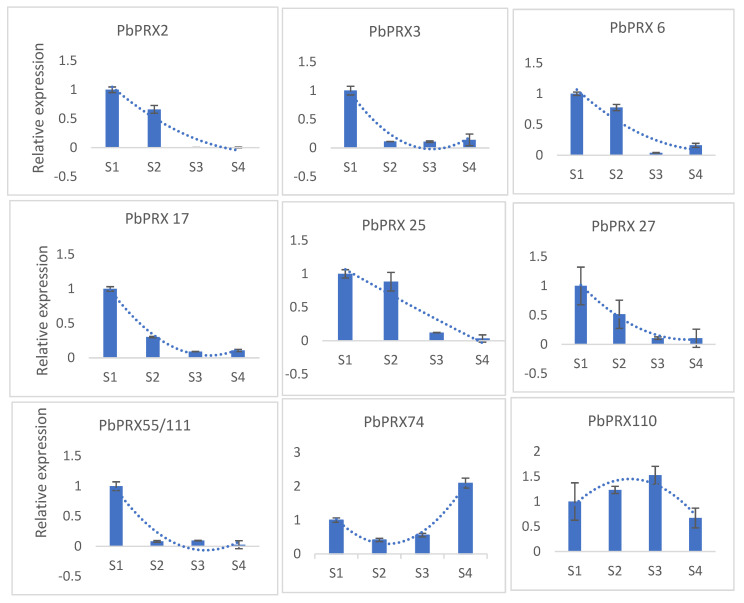
Expression levels of some particular *PbPRX* gene of “Dangshansu” pear fruit S1:15 days after full bloom (DAFB); S2:36DAFB; S3:79DAFB; S4:107DAFB.

On the basis of the RNA-seq database which combines the content of stone cells and lignin, we found that the expression levels of nine *PRX* (*PbPRX2, PbPRX3*, *PbPRX6*, *PbPRX17*, *PbPRX25*, *PbPRX27*, *PbPRX53*, *PbPRX55*, and *PbPRX110*) genes were closely related to lignin formation during pear fruit development, and may play a more important role than other genes. In order to verify whether these genes were associated with lignin content during pear fruit development, the expression levels of these eleven genes were analyzed by qRT-PCR. We chose the first four stages of pear fruit development which lignin is mostly formed in. Finally, the results of the qRT-PCR analysis indicated that the expression levels of all these nine *PRX* genes are closely tied with the content change of lignin during pear fruit development (Figure 7), none of them were different from RNA-seq data, supporting the reliability of our RNA-seq data.

## 4. Discussion

Class III peroxidases are plant-specific enzymes that play key roles in the responses to biotic and abiotic stress during plant growth and development, as well as being involved in plant lignification. While systematic and comprehensive whole-genome analyses of *PRX* gene families in *Arabidopsis thaliana*, *Oryza sativa,* and *Populus trichocarpa* have been reported [7,8,9], a systematic whole-genome study of this family has not previously been reported in pears. The full pear genome sequence serves as a useful tool for analyzing the pear *PRX* gene family to predict its evolutionary history and function [19]. In this study, we performed a comprehensive analysis of the *PRX* family genes in pears, including analysis of their phylogeny, gene structures, conserved motifs, chromosomal locations, gene duplication, and expression profiles. The number of *PRX* genes in pear (114) is higher than that in Arabidopsis (73) and Poplar (93) but slightly lower than that in rice (138), similar to that in maize(119), which indicates that the *PRX* genes in pear have expanded compared to those in *Arabidopsis* and *poplar*. Gene duplications are one of the primary driving forces in the evolution of genomes and genetic systems [33]. An increasing number of studies have shown that segmental duplication was largely responsible for the expansion of pear gene families. In this study, the number of *PbPRX* genes involved in segmental duplication is much more than that involved in tandem duplication, suggesting that segmental duplications are the main contributors to the expansion of the pear *PRX* family. By contrast, tandem duplication has contributed significantly to the expansion of this gene family in poplar [8]. According to the above analysis, we speculate that the expansion of the *PRX* gene family differed between monocotyledons and eudicotyledons. The Ka/Ks ratios of the 26 duplicated pairs show that purifying selection may be largely responsible for maintaining the functions of pear PRX proteins.

Phylogenetic analysis of the *PbPRX* gene family revealed that the exon/intron structures and motif compositions of these genes are relatively conserved. The 114 *PbPRX* genes contain different numbers of introns/exons, with most *PRX* genes containing more than two introns, indicating that there is some diversity in the pear *PRX* gene family. It is well known that the structural diversity of genes drives the evolution of multigene families. Furthermore, the differences in these characteristics detected between different subfamilies suggest that pear *PRX* members are functionally diversified.

Many studies have shown that introns were specifically inserted into plants and were retained in the genome during the course of evolution [34]. Therefore, we speculate that introns were gained or lost from the *PRX* coding region in a subfamily-specific manner.

Furthermore, MEME analysis revealed that different conserved motifs are present in each of the pear PRX proteins. However, some motifs with unknown functions are present in nearly every subgroup, these motifs might play important roles in the *PbPRX* family.

PRXs can catalyze the reduction of H_2_O_2_ by moving electrons to it and receiving electrons from various donor molecules. and are involved in several important physiological and developmental processes, including lignin and suberin formation, the cross-linking of cell wall components, wound healing, the removal of H_2_O_2_, the oxidation of toxic reductants, and defense against pathogen or insect attack. The result of Gene Ontology (GO) analysis indicates that ‘catalytic activity’, ‘antioxidant activity’, and ‘response to stimulus’ are the main functions of the *PbPRX* gene family, which corresponds to the acknowledged function of the peroxidase enzyme.

Gene expression patterns can provide important clues about gene function. We used publicly available genome-wide transcript profiling data from pear tissues as a resource to investigate the expression patterns of *PbPRXs* [19]. Of the 114 *PbPRX* genes, only 64 PbPRXs are expressed in fruit. According to the hierarchical cluster, 64 *PbPRXs* can be divided into ABCD four groups. We speculate that there are functional divergences in the four groups. *PRX* genes in Group A and B are smainly expressed at the first three stages of fruit development, which are major stages of lignification. So we speculate these genes are more important in lignification.

The content of stone cells in our study reached a peak at 49 DAFB, which agrees with the study carried out by Tao etc. [10]. The content of lignin increased at the earlier stage, the peak value was 35 DAFB, which is ten days earlier than that of stone cells. As is known to all, Stone cells mainly consist of lignin and cellulose. Lignin was accumulating in the cell wall, the secondary cell wall was then thickened constantly and eventually turned into stone cells, so that the content of stone cells reached a maximum after ten days. According to the RNA-seq database and qPCR, we found that *PbPRX2*, *PbPRX3*, *PbPRX6*, *PbPRX17*, *PbPRX25*, *PbPRX27*, and *PbPRX55* are likely to be involved in the lignification of pear stone cells.

## 5. Conclusions

In summary, the *PRX* family contains a large group of genes with essential functions in various developmental processes in plants. This study provides a foundation for further studying the functions of *PbPRX* genes, particularly for members with potentially important functions in lignification. However, further experiments should be conducted to directly examine the functions of *PbPRX* genes and their potential regulatory factors, including external cultivation and internal genetic factors.

## Figures and Tables

**Table 1:** Structural and biochemical information of members of *PbPRX* gene family in pear.

Gene Name	Gene ID	Chr(Mbp) ^z^	Stra-Nd ^y^	Start	End	CDS Length(bp)	Genomic Sequence Length(Bp)	Protein Length(aa)	Molecular Weight(da)	Pi ^x^	Exon Number
*PbPRX1*	Pbr032785.1	Chr1(10.7)	−	8171107	8172424	906	1318	302	32,907.3	8.13	3
*PbPRX2*	Pbr040489.1	Chr2(22.1)	+	15570372	15572304	996	1933	332	36,405.6	9.33	4
*PbPRX3*	Pbr035186.1	Chr2(22.1)	−	12486774	12488911	978	2138	326	35,266.2	8.82	4
*PbPRX4*	Pbr023311.1	Chr2(22.1)	+	16007519	16009267	855	1749	285	31,612.2	9.04	9
*PbPRX5*	Pbr003171.1	Chr2(22.1)	+	22018927	22019388	312	462	104	11,184.8	7.85	2
*PbPRX6*	Pbr000691.1	Chr3(27.4)	+	19118330	19120264	1053	1935	351	37,093.4	4.48	4
*PbPRX7*	Pbr013075.1	Chr3(27.4)	+	22733308	22735595	984	2288	328	35,036.7	9.29	4
*PbPRX8*	Pbr000689.1	Chr3(27.4)	+	19105701	19108029	1053	2329	351	37,452.8	4.29	4
*PbPRX9*	Pbr000686.1	Chr3(27.4)	+	19061527	19064600	999	3074	333	35,773.7	8.36	4
*PbPRX10*	Pbr013078.1	Chr3(27.4)	+	22708298	22709659	960	1362	320	34,387.9	6.23	4
*PbPRX11*	Pbr000687.1	Chr3(27.4)	+	19071289	19073019	975	1731	325	34,720.2	4.48	4
*PbPRX12*	Pbr013077.1	Chr3(27.4)	−	22711452	22712846	993	1395	331	35,887.7	6.59	3
*PbPRX13*	Pbr003832.1	Chr3(27.4)	−	26454998	26456517	999	1520	333	35,669.6	6.88	3
*PbPRX14*	Pbr022808.1	Chr3(27.4)	+	1626762	1627999	945	1238	315	34,167.4	5.61	3
*PbPRX15*	Pbr013214.1	Chr3(27.4)	−	21696976	21698605	1026	1630	342	37,353.7	4.81	4
*PbPRX16*	Pbr021747.1	Chr3(27.4)	−	12196399	12198178	1065	1780	355	undefined	undefined	4
*PbPRX17*	Pbr033934.1	Chr3(27.4)	−	24516702	24522508	1059	5807	353	38,476.8	5.62	12
*PbPRX18*	Pbr022809.1	Chr3(27.4)	+	1639130	1639417	288	288	96	10,491.1	7.78	1
*PbPRX19*	Pbr032800.1	Chr4(13.4)	−	11190141	11192827	1026	2687	342	38,505.1	8.93	4
*PbPRX20*	Pbr006566.1	Chr4(13.4)	+	816311	817595	462	1285	154	16,924.5	9.47	3
*PbPRX21*	Pbr002542.1	Chr5(28.4)	+	22011532	22012678	1041	1147	347	38,598.6	9.05	2
*PbPRX22*	Pbr041097.1	Chr5(28.4)	+	20588048	20590614	1218	2567	406	44,469.3	6.03	4
*PbPRX23*	Pbr002505.1	Chr5(28.4)	−	21659712	21661441	1059	1730	353	39,178.2	5.91	3
*PbPRX24*	Pbr000438.1	Chr5(28.4)	+	25395691	25397689	1176	1999	392	42,972.1	9.22	5
*PbPRX25*	Pbr000146.1	Chr5(28.4)	−	27528334	27530297	1026	1964	342	38,740.2	8.30	4
*PbPRX26*	Pbr013845.1	Chr6(23.1)	+	19145761	19152732	2607	6972	869	98,120.6	6.33	11
*PbPRX27*	Pbr014180.2	Chr6(23.1)	+	8740620	8743196	648	2577	216	23,389.3	5.11	8
*PbPRX28*	Pbr002948.1	Chr7(15.3)	−	12585545	12589315	1416	3771	472	51,545.7	8.99	6
*PbPRX29*	Pbr010975.1	Chr7(15.3)	+	11895741	11897397	990	1657	330	35,665.4	9.65	4
*PbPRX30*	Pbr002950.1	Chr7(15.3)	+	12603529	12604962	957	1434	319	34,580.5	9.31	3
*PbPRX31*	Pbr002956.1	Chr7(15.3)	+	12644802	12646457	990	1656	330	35,812.7	9.73	4
*PbPRX32*	Pbr010973.1	Chr7(15.3)	−	11880070	11881667	957	1598	319	34,607.6	9.43	3
*PbPRX33*	Pbr040033.1	Chr7(15.3)	−	14720194	14721588	987	1395	329	35,549.3	9.02	4
*PbPRX34*	Pbr013905.1	Chr7(15.3)	−	12981008	12982502	1011	1495	337	37,239.5	8.72	3
*PbPRX35*	Pbr010977.1	Chr7(15.3)	+	11900730	11901663	486	934	162	18,183.7	9.11	3
*PbPRX36*	Pbr002947.1	Chr7(15.3)	−	12582679	12583729	330	1051	110	12,014.7	5.34	2
*PbPRX37*	Pbr002957.1	Chr7(15.3)	+	12650388	12650724	252	337	84	9,017.3	6.54	2
*PbPRX38*	Pbr010976.1	Chr7(15.3)	+	11897792	11898914	429	1123	143	16,177.7	8.44	2
*PbPRX39*	Pbr026505.1	Chr8(17.1)	−	4426466	4427581	996	1116	332	35,928.0	8.79	2
*PbPRX40*	Pbr026503.1	Chr8(17.1)	+	4440333	4442618	996	2286	332	36,048.0	8.62	2
*PbPRX41*	Pbr026502.1	Chr8(17.1)	+	4444100	4445303	984	1204	328	35,583.5	8.57	2
*PbPRX42*	Pbr026504.1	Chr8(17.1)	−	4434098	4435213	996	1116	332	36,043.2	8.89	2
*PbPRX43*	Pbr036549.1	Chr8(17.1)	−	16020359	16022020	1014	1662	338	36,117.8	5.44	4
*PbPRX44*	Pbr006119.1	Chr8(17.1)	−	15274439	15276078	966	1640	322	34,724.7	8.41	4
*PbPRX45*	Pbr036474.1	Chr8(17.1)	+	16467636	16469367	972	1732	324	34,752.3	5.32	5
*PbPRX46*	Pbr006117.1	Chr8(17.1)	−	15269333	15270972	966	1640	322	34,697.6	8.11	4
*PbPRX47*	Pbr004299.1	Chr8(17.1)	+	6081866	6083665	933	1800	311	33,396.9	4.82	4
*PbPRX48*	Pbr020588.1	Chr8(17.1)	−	9421357	9424517	972	3161	324	34,409.4	5.88	10
*PbPRX49*	Pbr020590.1	Chr8(17.1)	+	9511517	9514687	972	3171	324	34,409.4	5.88	10
*PbPRX50*	Pbr005400.1	Chr9(22.4)	+	5466505	5468645	957	2141	319	34,371.0	8.71	3
*PbPRX51*	Pbr018082.1	Chr9(22.4)	+	16626484	16628070	984	1587	328	35,481.6	9.08	4
*PbPRX52*	Pbr018080.1	Chr9(22.4)	+	16633205	16634674	981	1470	327	35,595.7	9.01	4
*PbPRX53*	Pbr026235.1	Chr9(22.4)	+	20559304	20560595	984	1292	328	35,943.9	8.58	1
*PbPRX54*	Pbr019188.1	Chr9(22.4)	+	10558578	10565436	2637	6859	879	97,910.3	5.85	16
*PbPRX55*	Pbr027164.1	Chr10(26.2)	+	24310314	24313743	1053	3430	351	38,056.8	5.20	3
*PbPRX56*	Pbr010270.1	Chr10(26.2)	−	1153171	1154446	1041	1276	347	38,616.7	9.26	2
*PbPRX57*	Pbr010258.1	Chr10(26.2)	+	1043826	1048256	1497	4431	499	56,140.4	8.20	3
*PbPRX58*	Pbr010213.1	Chr10(26.2)	−	612484	614210	1071	1727	357	39,612.6	5.44	4
*PbPRX59*	Pbr031894.1	Chr10(26.2)	−	5317405	5319983	1011	2579	337	38,342.9	8.30	4
*PbPRX60*	Pbr020725.1	Chr10(26.2)	−	17517628	17522171	789	4544	263	28,676.3	6.51	8
*PbPRX61*	Pbr020734.1	Chr10(26.2)	+	17377042	17381585	789	4544	263	28,676.3	6.51	8
*PbPRX62*	Pbr003308.1	Chr11(30.3)	−	22280347	22282143	999	1797	333	35,706.4	7.53	4
*PbPRX63*	Pbr011562.1	Chr11(30.3)	+	25372571	25375151	945	2581	315	33,541.9	9.35	4
*PbPRX64*	Pbr011557.1	Chr11(30.3)	+	25338613	25347124	3945	8512	1315	143,415.6	5.78	18
*PbPRX65*	Pbr003309.1	Chr11(30.3)	−	22273464	22275146	975	1683	325	347,76.3	4.57	4
*PbPRX66*	Pbr011560.1	Chr11(30.3)	+	25362475	25363817	969	1343	323	35,081.3	9.47	3
*PbPRX67*	Pbr022326.1	Chr11(30.3)	−	6078338	6082106	906	3769	302	32,772.3	7.54	4
*PbPRX68*	Pbr011559.1	Chr11(30.3)	+	25356556	25357860	963	1305	321	34,899.8	8.97	3
*PbPRX69*	Pbr003310.1	Chr11(30.3)	−	22266804	22267913	744	1110	248	26,540.9	4.38	3
*PbPRX70*	Pbr035815.1	Chr12(22.8)	+	16715336	16718714	1017	3379	339	35,696.5	5.10	4
*PbPRX71*	Pbr014607.1	Chr12(22.8)	−	4710343	4713291	927	2949	309	32,685.0	8.91	4
*PbPRX72*	Pbr026058.1	Chr12(22.8)	+	3759751	3760860	936	1110	312	33,748.3	8.09	2
*PbPRX73*	Pbr014605.1	Chr12(22.8)	−	4732591	4733625	750	1035	250	26,220.3	8.94	3
*PbPRX74*	Pbr008291.1	Chr12(22.8)	−	9391476	9394142	753	2667	251	27,632.2	5.30	9
*PbPRX75*	Pbr008320.1	Chr12(22.8)	+	9106502	9107578	471	1077	157	17,355.4	5.82	2
*PbPRX76*	Pbr035513.1	Chr12(22.8)	+	22606333	22607214	483	882	161	17,774.6	8.75	2
*PbPRX77*	Pbr034800.1	Chr13(15.1)	+	14344175	14345739	957	1565	319	34,329.0	9.37	4
*PbPRX78*	Pbr034821.1	Chr13(15.1)	+	14483716	14487956	1722	4241	574	63,176.3	6.96	17
*PbPRX79*	Pbr015016.1	Chr13(15.1)	−	8716264	8717618	1002	1355	334	35,742.2	4.42	4
*PbPRX80*	Pbr030045.1	Chr13(15.1)	−	4024846	4026504	1017	1659	339	36,936.2	4.81	4
*PbPRX81*	Pbr014793.1	Chr13(15.1)	−	11876947	11878622	996	1676	332	36,098.2	7.03	4
*PbPRX82*	Pbr015032.1	Chr13(15.1)	−	8833969	8835416	975	1448	325	35,052.1	5.49	3
*PbPRX83*	Pbr039193.1	Chr13(15.1)	−	3561750	3562436	687	687	229	25,105.6	8.93	1
*PbPRX84*	Pbr016853.1	Chr14(20.3)	−	3626045	3627070	834	1026	278	29,540.2	6.41	3
*PbPRX85*	Pbr005912.1	Chr15(43.6)	+	2978373	2985696	2595	7324	865	93,865.2	7.87	16
*PbPRX86*	Pbr007872.1	Chr15(43.6)	−	20261904	20266643	1020	4740	340	37,262.1	5.36	5
*PbPRX87*	Pbr002672.1	Chr15(43.6)	−	1214104	1216253	984	2150	328	36,549.0	9.52	4
*PbPRX88*	Pbr009308.1	Chr15(43.6)	−	3677275	3679424	984	2150	328	36,549.0	9.52	4
*PbPRX89*	Pbr010632.1	Chr15(43.6)	+	12931761	12933817	963	2057	321	34,691.7	7.53	4
*PbPRX90*	Pbr027845.1	Chr15(43.6)	−	9981997	9985179	993	3183	331	35,362.0	5.40	10
*PbPRX91*	Pbr042913.1	Chr15(43.6)	−	36603917	36607371	1035	3455	345	37,741.8	6.26	11
*PbPRX92*	Pbr006005.1	Chr16(20.6)	−	10236903	10238012	936	1110	312	33,748.3	8.09	2
*PbPRX93*	Pbr036153.1	Chr16(20.6)	+	62244	64019	1011	1776	337	36,758.4	8.03	2
*PbPRX94*	Pbr036152.1	Chr16(20.6)	+	59021	60571	1023	1551	341	37,562.1	8.28	2
*PbPRX95*	Pbr011189.1	Chr16(20.6)	−	6703300	6706908	1341	3609	447	49,287.0	9.10	5
*PbPRX96*	Pbr034488.2	Chr17(25.3)	−	22537048	22538599	978	1552	326	35,455.4	8.78	4
*PbPRX97*	Pbr034480.1	Chr17(25.3)	−	22583700	22585282	981	1583	327	35,426.4	9.01	4
*PbPRX98*	Pbr026772.1	Chr17(25.3)	−	7814591	7816038	975	1448	325	35,052.1	5.49	3
*PbPRX99*	Pbr034479.1	Chr17(25.3)	−	22587987	22589313	600	1327	200	21,632.4	9.41	5
*PbPRX100*	Pbr006343.1	scaffold132.0.1(0.8)	−	817092	818160	876	1069	292	32,297.8	5.06	3
*PbPRX101*	Pbr015968.1	scaffold235.0(0.6)	−	499783	502085	1035	2303	345	37,911.4	5.25	4
*PbPRX102*	Pbr015965.1	scaffold235.0(0.6)	+	472592	481134	876	8543	292	31,040.9	7.55	4
*PbPRX103*	Pbr015969.1	scaffold235.0(0.6)	−	513208	513655	330	448	110	11,818.5	5.10	2
*PbPRX104*	Pbr027136.1	scaffold440.0(0.4)	−	95386	98157	1308	2772	436	47,426.7	7.70	11
*PbPRX105*	Pbr027137.1	scaffold440.0(0.4)	+	194284	197055	1245	2772	415	45,080.0	7.71	11
*PbPRX106*	Pbr037664.1	scaffold770.0(0.2)	−	44241	46306	888	2066	296	31,950.3	7.53	4
*PbPRX107*	Pbr037665.1	scaffold770.0(0.2)	+	152615	154680	888	2066	296	31,950.3	7.53	4
*PbPRX108*	Pbr037526.1	scaffold766.0(0.2)	−	279	1961	990	1683	330	36,383.7	5.91	3
*PbPRX109*	Pbr041827.1	scaffold955.0(0.1)	−	120167	121688	981	1522	327	35,732.6	8.82	4
*PbPRX110*	Pbr000988.3	scaffold1008.0(0.1)	+	122239	124533	768	2295	256	27,802.6	5.82	9
*PbPRX111*	Pbr007903.1	scaffold1455.0(0.04)	+	21962	25441	1053	3480	351	38,098.9	5.20	3
*PbPRX112*	Pbr007909.1	scaffold1459.0(0.04)	+	4474	5451	978	978	326	36,027.2	8.33	1
*PbPRX113*	Pbr007908.1	scaffold1459.0(0.04)	+	1560	3254	978	1695	326	35,817.9	6.00	1
*PbPRX114*	Pbr008699.1	scaffold1534.0(0.03)	−	10892	13612	1353	2721	451	49,351.1	5.63	10

^z^ On which chromosome every gene is located and the length of the chromosome. ^y^ Forward or reverse of gene on chromosome. ^x^ Isoelectric point of every protein.

**Table 2:** Major MEME motif sequences in pear PRX proteins.

Motif	Width	Seqs ^z^	E-Value	Conserved Amino Acid Sequences
1	21	78	9.8 × 10^−1252^	LLRMHFHDCFVQGCDASVLLD
2	21	96	1.0 × 10^−895^	HTIGQARCTTFRARIYNETNI
3	15	95	2.0 × 10^−883^	CPGVVSCADILAIAA
4	15	101	8.0 × 10^−729^	TGGPTWKVPTGRRDG
5	15	103	1.7 × 10^−648^	FFQQFAKSMVKMGNI
6	32	91	1.4 × 10^−1208^	FDNSYFKNLIQKKGLLHSDQQLFNGGSTDSIV
7	21	81	5.5 × 10^−730^	PNNNSLRGFEVVDKIKSQVEK
8	15	91	5.5 × 10^−632^	TGSNGEIRKNCRVVN
9	21	79	7.7 × 10^−611^	GFYSRTCPTAESIVKQTVQTH
10	15	100	1.9 × 10^−581^	SHGLSQTDMVALSGA
11	21	101	1.4 × 10^−382^	ANETINLPAPTFNVSQLIQSF
12	15	88	4.7 × 10^−251^	NKTYATQLQQMCPKN
13	57	8	2.1 × 10^−180^	KTKTGGPFGTMRCPAEQAHGANNGLDIAVRLLEPIKQQFPILSYA DFYQLAGVVAVE
14	29	12	6.5 × 10^−132^	RRDLRALIYSKNCAPIMLRIAWHDAGTYD
15	11	59	1.9 × 10^−132^	DTPNFTGEKTA
16	15	34	8.6 × 10^−109^	GGDNNLSPLDVTSPT
17	21	9	3.0 × 10^−93^	HTLGRCHKERSGFEGPWTPNP
18	29	12	8.3 × 10^−117^	EGLLKLPTDKALLDDPEFRLYVELYAKDE
19	21	9	2.5 × 10^−51^	PDNLSLAGDGFDTVIKAKAAV
20	6	81	1.5 × 10^−49^	RDSVVL

^z^ Total number of proteins which have the special motif of *PRX*s gene family.

**Table 3:** Duplicated gene pairs, Ka, Ks value, and duplicate type of *PbPRX* gene family.

Paralogous Pairs	Ka	Ks	Ka/Ks	Purifying Selection	Duplicate Type
*PbPRX1-PbPRX34*	0.06607	0.20824	0.31728	Yes	Segmental (WGD)
*PbPRX2-PbPRX85*	0.36046	2.11212	0.17067	Yes	Segmental(WGD)
*PbPRX4-PbPRX78*	0.12760	0.36480	0.34977	Yes	Segmental(WGD)
*PbPRX5-PbPRX89*	0.29346	0.66614	0.44053	Yes	Segmental(WGD)
*PbPRX7-PbPRX29*	0.21605	1.82632	0.11830	Yes	Segmental(WGD)
*PbPRX9-PbPRX62*	0.06251	0.14092	0.44361	Yes	Segmental(WGD)
*PbPRX11-PbPRX69*	0.03789	0.13969	0.27123	Yes	Segmental(WGD)
*PbPRX12-PbPRX68*	0.21650	1.21569	0.17809	Yes	Segmental(WGD)
*PbPRX14-PbPRX50*	0.18371	0.66916	0.27454	Yes	Segmental(WGD)
*PbPRX15-PbPRX80*	0.04732	0.15792	0.29963	Yes	Segmental(WGD)
*PbPRX17-PbPRX27*	0.03944	0.27059	0.14576	Yes	Segmental(WGD)
*PbPRX17-PbPRX74*	0.11130	1.33285	0.08350	Yes	Segmental(WGD)
*PbPRX21-PbPRX56*	0.03789	0.28908	0.13109	Yes	Segmental(WGD)
*PbPRX21-PbPRX58*	0.34305	2.24266	0.15297	Yes	Segmental(WGD)
*PbPRX23-PbPRX58*	0.04549	0.09761	0.46601	Yes	Segmental(WGD)
*PbPRX25-PbPRX59*	0.00946	0.13415	0.07055	Yes	Segmental(WGD)
*PbPRX28-PbPRX31*	0.00600	0.01078	0.55631	Yes	Segmental(WGD)
*PbPRX28-PbPRX38*	0.12609	0.18468	0.68272	Yes	Segmental(WGD)
*PbPRX30-PbPRX32*	0.00414	0.00882	0.46968	Yes	Segmental(WGD)
*PbPRX36-PbPRX37*	0.20381	0.21673	0.94039	Yes	Segmental(WGD)
*PbPRX51-PbPRX97*	0.45986	4.06384	0.11316	Yes	Segmental(WGD)
*PbPRX53-PbPRX83*	0.38368	0.75805	0.50614	Yes	Segmental(WGD)
*PbPRX56-PbPRX57*	0.01007	0.02564	0.39263	Yes	Segmental(WGD)
*PbPRX69-PbPRX70*	0.26718	3.83175	0.06973	Yes	Segmental(WGD)
*PbPRX71-PbPRX102*	0.15772	0.35349	0.44617	Yes	Segmental(WGD)
*PbPRX81-PbPRX95*	0.07174	0.16084	0.44603	Yes	Segmental(WGD)

## Data Availability

All data, tables, figures, and results in this paper are our own and original.

## Supplementary Materials

The following are available online at https://www.mdpi.com/article/10.3390/plants10091874/s1, Table S1 List of *PbPRX* and internal control genes primers used for qPCR gene expression.

## References

[1] Cosio C., Dunand C. (2009). Specific functions of individual class III peroxidase genes. J. Exp. Bot..

[2] Bindschedler L.V., Dewdney J., Blee K.A., Stone J.M., Asai T., Plotnikov J., Denoux C., Hayes T., Gerrish C., Davies D.R. (2006). Peroxidase-dependent apoplastic oxidative burst in *Arabidopsis* required for pathogen resistance. Plant J..

[3] Daudi A.Z., Cheng J.A., Brien O., Mammarella N., Khan S., Ausubel F.M., Bolwell G.P. (2012). The apoplastic oxidative burst peroxidase in *Arabidopsis* is a major component of pattern-triggered immunity. Plant Cell.

[4] Herrero J., Fernández-Pérez F., Yebra T., Novo-Uzal E., Pomar F., Pedreo M., Cuello J., Guéra A., Esteban-Carrasco A., Zapata J.M. (2013). Bioinformatic and functional characterization of the basic peroxidase 72 from *Arabidopsis* thaliana involved in lignin biosynthesis. Planta.

[5] Welinder K.G. (1992). Plant peroxidases: Structure–function relationships. Plant Peroxidases.

[6] Welinder K.G. (1992). Superfamily of plant, fungal and bacterial peroxidases. Curr. Opin. Struct. Biol..

[7] Passardi F., Longet D., Penel C., Dunand C. (2004). The class III peroxidase multigenic family in rice and its evolution in land plants. Phytochemistry.

[8] Ren L., Liu Y., Qian T., Qi L., Wang X., Zeng Q. (2014). Subcellular relocalization and positive selection play key roles in the retention of duplicate genes of populus class III peroxidase family. Plant Cell.

[9] Tognolli M., Penel C., Greppin H., Simon P. (2002). Analysis and expression of the class III peroxidase large gene family in *Arabidopsis* thaliana. Gene.

[10] Tao S.T. (2009). Characterization of Sclereid Structure and Composition and Cloning of Sclereid Related Enzyme Genes in Pear (*Pyrus*) Fruit. Ph.D. Thesis.

[11] Tao S.T., Khanizadeh S., Zhang H., Zhang S.L. (2009). Anatomy, ultrastructure and lignin distribution of stone cells in two *Pyrus species*. Plant Sci..

[12] Van de Peer Y., Meyer A. (2005). Large-scale gene and ancient genome duplications. Evol. Genome.

[13] Fawcett J.A., Maereand S., Van de Peer Y. (2009). Plants with double genomes might have had a better chance to survive the Cretaceous-Tertiary extinction event. Proc. Natl. Acad. Sci. USA.

[14] Velasco R., Zharkikh A., Affourtit J., Dhingra A. (2010). The genome of the domesticated apple (Malus × domestica Borkh). Nat. Genet..

[15] Taylor J.S., Raes J. (2004). Duplication and divergence: The evolution of new genes and old ideas. Annu. Rev. Genet..

[16] Yu J., Wang J., Lin W., Song G., Li H., Zhou J., Ni P., Dong W. (2005). The Genomes of Oryza sativa: A History of Duplications. PLoS Biol..

[17] Sharoni A.M., Nuruzzaman M., Satoh K., Shimizu T., Kondoh H., Sasaya T., Choi I., Omura T., Kikuchi S. (2011). Gene Structures, Classification and Expression Models of the AP2/EREBP Transcription Factor Family in Rice. Plant Cell Physiol..

[18] Zhao Y.X., Cai M.J., Zhang X.B., Li Y.R., Zhang J.H., Zhao H.L., Kong F., Zheng Y.L., Qiu F.Z. (2014). Genome-Wide Identification, Evolution and Expression Analysis of mTERFGene Family in Maize. PLoS ONE.

[19] Wu J., Wang Z.W., Shi Z.B., Zhang S., Ming R., Zhu S.L., Khan M.A., Tao S.T., Korban S.S., Wang H. (2013). The genome of the pear (*Pyrus bretschneideri Rehd.*). Genome Res..

[20] Finn R.D., Bateman A., Clements J., Coggill P., Eberhardt R.Y., Eddy S.R., Heger A., Hetherington K., Holm L., Mistry J. (2014). Pfam: The protein families database. Nucleic Acids Res..

[21] Letunic I., Doerks T., Bork P. (2012). SMART 7: Recent updates to the protein domain annotation resource. Nucleic Acids Res..

[22] Hu B., Jin J., Guo A., Zhang H., Luo J., Gao G. (2015). GSDS 2.0: An upgraded gene feature visualization server. Bioinformatics.

[23] Bailey T.L., Elkan C. (1995). Unsupervised Learning of Multiple Motifs in Biopolymers Using Expectation Maximization. Mach. Learn..

[24] Lee T.H., Tang H., Wang X., Paterson A.H. (2013). PGDD: A database of gene andgenome duplication in plants. Nucleic Acids Res..

[25] Wang Y., Tang H., DeBarry J.D., Tan X., Li J., Wang X., Lee Th Jin H., Marler B., Guo H. (2012). MCScanX: A toolkit for detection and evolutionary analysis of genesynteny and collinearity. Nucleic Acids Res..

[26] Wang D., Zhang Y., Zhang Z., Zhu J., Yu J. (2010). KaKs_Calculator 2.0: A Toolkit Incorporating Gamma-Series Methods and Sliding Window Strategies. Genom. Proteom. Bioinform..

[27] Zdobnov E.M., Apweiler R. (2001). InterProScan—An integration platform for the signature-recognition methods in InterPro. Bioinformatics.

[28] Harris M., Clark J., Ireland A., Lomax J., Ashburner M., Foulger R., Eilbeck K., Lewis S., Marshall B., Mungall C. (2004). The gene ontology (GO) database and informatics resource. Annu. Rev. Genet..

[29] Ye J., Fang L., Zheng H., Zhang Y., Chen J., Zhang Z., Wang J., Li S., Li R., Bolund L. (2006). WEGO: A web tool for plotting GO annotations. Nucleic Acids Res..

[30] Livak K.J., Schmittgen T.D. (2001). Analysis of Relative Gene Expression Data Using Real-Time Quantitative PCR and the ^2-D^DCT Method. Methods.

[31] Syros T., Yupsanis T., Zafiriadis H., Economou A. (2004). Activity and isoforms of peroxidases, lignin and anatomy, during adventitious rooting in cuttings of *Ebenus cretica* L. J. Plant Physiol..

[32] Cannon C.P., Braunwald E., McCabe C.H., Rader D.J., Rouleau J.L., Belder R., Joyal S.V., Hill K.A., Pfeffer M.A., Skene A.M. (2004). Intensive versus Moderate Lipid Lowering with Statins after Acute Coronary Syndromes. N. Engl. J. Med..

[33] Moore R.C., Purugganan M.D. (2003). The early stages of duplicate gene evolution. Proc. Natl. Acad. Sci. USA.

[34] Rogozin I.B., Wolf Y.I., Sorokin A.V., Mirkin B.G., Koonin E.V. (2003). Remarkable Interkingdom Conservation of Intron Positions and Massive, Lineage-Specific Intron Loss and Gain in Eukaryotic Evolution. Curr. Biol..

