# Supplementary material for: Analysis of PRX Gene Family and Its Function on Cell Lignification in Pears (Pyrus bretschneideri)
Source: Plants (Basel). 2021 Sep 10;10(9):1874. doi: 10.3390/plants10091874 (PMC8470002; doi:10.3390/plants10091874)
Supplement: Supplementary file 1 [file plants-10-01874-s001.zip › plants-1368417-supplementary.pdf]

Supplementary Table S1. List of *PbPRX* and internal control genes primers used for qPCR gene expression.

The primers used for amplifying PRX genes for Real-time PCR analysis		
PbPRX2	Forward	TCCTGCCCCAAACAACACAT
	Reverse	GTTGCCCGACTGGTTGTAGA
PbPRX3	Forward	GCCTTGTCAAAGATGCTGTCA
	Reverse	TGCCTTGTTGTTTGCAGTGG
PbPRX6	Forward	TGGGCGTGCTAGATGTCAAT
	Reverse	TCCAAATTGGTTAGCACACTGC
PbPRX17	Forward	ATTCAGCTGGAACTTACGACACC
	Reverse	TAGAAGTCAGCGTAGGAGAGGATG
PbPRX25	Forward	GCAATGAGCAGGATCCAGGT
	Reverse	CTGTCCATCTCCTTCTCAGACAAC
PbPRX27	Forward	CGGCACAGAAAGATGGCATT
	Reverse	TGCTTGATGGGCTCCAAGAG
PbPRX53	Forward	ATCTCCCAGCTCATCCAGGT
	Reverse	TGTAACTCGGGTCGAACTGC
PbPRX74	Forward	GAAGGTCGTTTGCCAGATGC
	Reverse	CACCTACCCAAGGTGTGTCC
PbPRX110	Forward	GATAAGCCGGAAAATCGGCTC
	Reverse	ACGAAGCATGAGAGGAGCAC
PbPRX111/PbPRX55	Forward	CGCAAGATGCTACCATGGAC
	Reverse	ACCTTGGCGGTTCATGAGAT
Actin	Forward	TACTCTTTCACCACAACTGC
	Reverse	CTCGTAACTCTTCTCCACAG
